# Is TIMP‐1 a biomarker for periodontal disease? A systematic review and meta‐analysis

**DOI:** 10.1111/jre.12957

**Published:** 2021-12-01

**Authors:** Puk de Brouwer, Floris J. Bikker, Henk S. Brand, Wendy E. Kaman

**Affiliations:** ^1^ Department of Oral Biochemistry Academic Centre for Dentistry Amsterdam University of Amsterdam and VU University Amsterdam Amsterdam Gustav Mahlerlaan Netherlands

**Keywords:** biomarker, gingivitis, periodontitis, TIMP‐1, tissue inhibitor of metalloproteases

## Abstract

**Objective:**

One of the most important families of proteases associated with periodontal disease is the family of the matrix metalloproteinases (MMPs). Their activity is regulated by tissue inhibitors of metalloproteinases (TIMPs), and an imbalance between MMP activity and regulation by TIMPs has been associated with the progression of periodontal disease. This strong interaction between TIMPs and MMPs might be an indication that TIMPs can be used as a biomarker to monitor periodontal disease progression in oral fluids. In particular, TIMP‐1 is a frequently studied biomarker for periodontal diseases. Therefore, the aim of this systematic review was to evaluate the scientific literature regarding TIMP‐1 concentrations in oral fluids of patients suffering from periodontitis or gingivitis in comparison to healthy individuals.

**Material and Methods:**

PubMed/ MedLine and Web of Science databases were searched electronically. Studies that met the inclusion criteria were systematically evaluated and assessed for eligibility and risk of bias. Meta‐analysis was performed through the random effects model to assess the association between periodontitis/gingivitis and TIMP‐1 concentration in stimulated saliva, unstimulated saliva, and gingival crevicular fluid (GCF).

**Results:**

The search strategy provided a total of 322 studies of which 10 studies met all inclusion criteria. Two studies investigated TIMP‐1 concentrations in GCF, three studies in unstimulated saliva, and five studies investigated TIMP‐1 concentrations in stimulated saliva. Three studies revealed that TIMP‐1 levels in oral fluids were significantly decreased in periodontal disease. Meta‐analysis revealed that there is no statistically significant difference between TIMP‐1 concentration in oral fluids of periodontitis/gingivitis patients in comparison to healthy individuals.

**Conclusions:**

This systematic review with meta‐analysis shows that periodontal diseases are not associated with a statistically significant change in TIMP‐1 concentration in oral fluids.

## 1 INTRODUCTION

Degradation of periodontal tissue is related to the activity of proteases involved in the inflammatory process.^1^, ^2^ One of the most important families of proteases associated with periodontal disease are the matrix metalloproteinases (MMPs).^3^ In particular, MMP‐8, MMP‐9, and, to a lesser extent, MMP‐14 have been studied in relation to periodontitis.^4^ These MMPs are not only responsible for the degradation of the extracellular matrix during periodontitis but are also key factors in tissue remodeling processes.^5^, ^6^ The activity of MMPs is regulated by tissue inhibitors of metalloproteinases (TIMPs) which are produced and secreted by many cell types. Their production is regulated by various cytokines and growth factors. Besides MMPs, TIMPs also regulate the activity of other families such as the disintegrin metalloproteinases (ADAM and ADAMTS).^7^ Therefore, TIMPs play a crucial role in important biological processes like the formation of the extracellular matrix and cell proliferation.

Upon binding to MMPs, TIMPs act like a wedge which connects to the active site of the MMP and thereby blocking the binding of substrate to MMP, resulting in reduced MMP activity.^7^ An imbalance between MMP activity and regulation by TIMP has been associated with progression of periodontal disease. This imbalance results in the degradation of matrix proteins, and thereby contributes to the destruction of periodontal tissue.^8^, ^9^, ^10^, ^11^

The strong relation between TIMPs and MMPs suggests that TIMPs might potentially serve as a biomarker to diagnose periodontitis and monitor disease progression in oral fluids.^12^, ^13^ Of the four types of TIMPs identified in humans, TIMP‐1, an inhibitor of MMP‐9, has most often been associated with periodontal disease. However, so far the diagnostic value of TIMP‐1 in periodontal disease has not been systematically reviewed.^7^

In this context, the aim of this systematic review was to analyze the validity of TIMP‐1 solely as a biomarker to diagnose periodontal disease in saliva and gingival crevicular fluid (GCF).

## 2 MATERIALS AND METHODS

This systematic review was elaborated according to the Preferred Reporting Items for Systematic Reviews and Meta‐Analysis (PRISMA) guidelines.^14^ The PRISMA checklist is included in Table S1.^15^ The protocol was registered at the International Prospective Register of Systematic Reviews (PROSPERO) under registration number CRD42021246024.^16^

### 2.1 Research strategy, selection, and inclusion and exclusion criteria

An electronic database search was performed until December 31^th^ 2020 in the database of the National Library of Medicine (MEDLINE by PubMed) and Web of Science using a combination of medical subject headings (MeSH) terms and free text words (Appendix S1).

**FIGURE 1:**
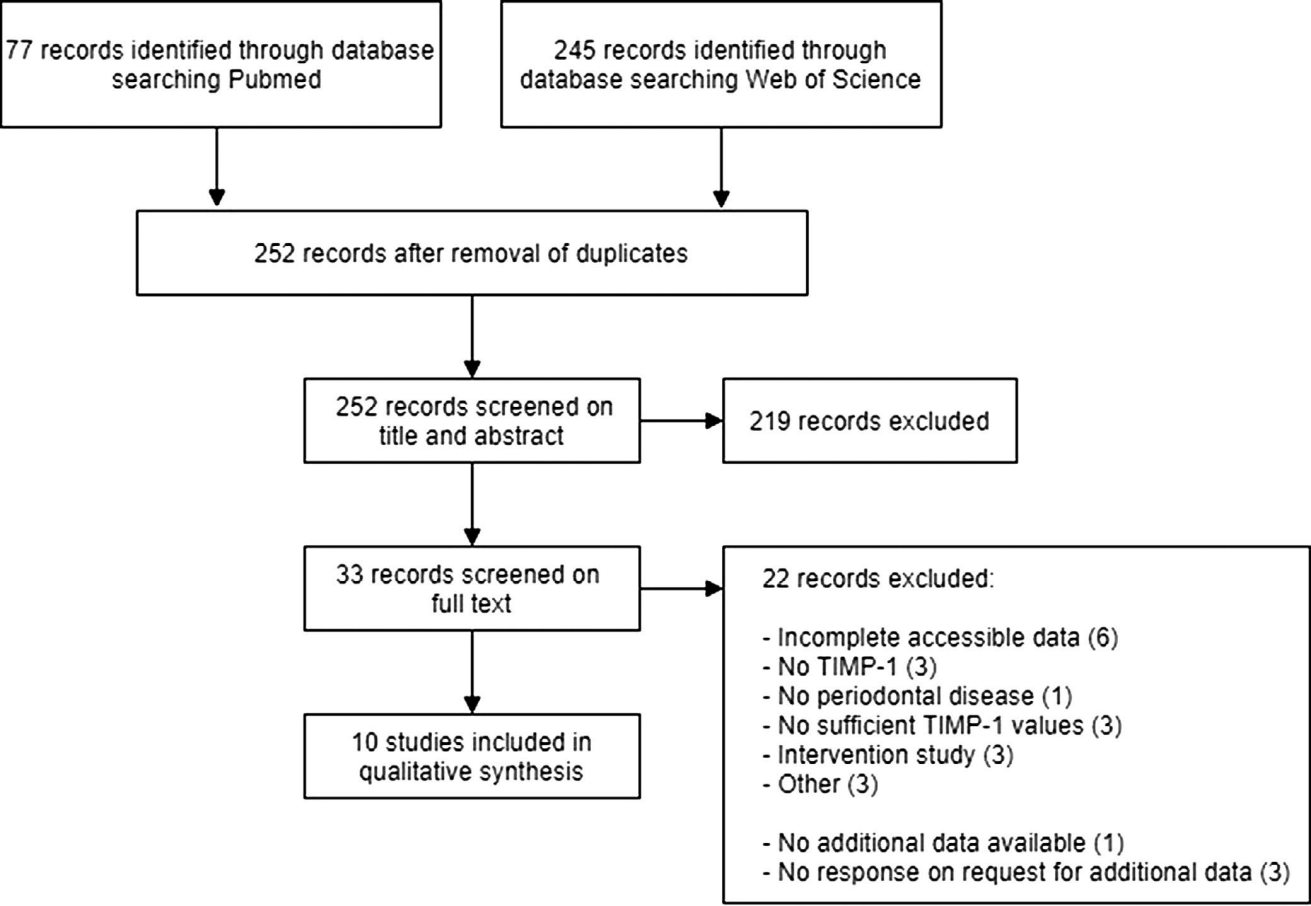
Schematic PRISMA diagram for procedural methodology

The resulting articles were reviewed independently by title, abstract, and full text by two reviewers (PdB and WEK). Any disagreements during the review process were resolved by discussion. Articles that met the following inclusion criteria were retrieved: studies including patients with chronic periodontitis or gingivitis diagnosed based on clinical parameters, publications in English, and studies investigating TIMP‐1 concentrations in oral fluids. Publications that did not present a compatible methodology for a systematic analysis were excluded (e.g., reviews, opinions, book chapters, abstracts, and editorial letters). In vitro studies, animal studies, experiments that interfered with the expression of TIMP‐1 through therapeutic methods, studies that evaluated patients with systemic diseases, studies that investigated other types of periodontitis than chronic periodontitis or gingivitis, studies investigating the systemic effect of proteases, studies that evaluated pregnant patients, and studies that evaluated children were also excluded. In addition, studies without a control group were also excluded. Where possible, sample sizes, mean‐values, and standard deviations were retrieved from the publications or calculated based on the available data. In case limited data were available, study investigators were contacted to retrieve the missing information. The whole process of literature selection was executed according to the PRISMA guidelines and is summarized in Figure 1.

### 2.2 Data extraction

Information retrieved from all studies involved: authors, year of publication, number of patients diagnosed with periodontitis and number of controls, severity of the periodontal disease, criteria for diagnosis used for inclusion, TIMP‐1 detection method, study results, and relevant conclusions.

### 2.3 Assessment of risk of bias

The selected studies were analyzed with tools from the National Heart, Lung, and Blood Institute (NHLBI) to assess their quality.^17^ First, the selected studies were classified by research design.^18^ Depending on the research design, the following three risk assessment questionnaire tools were used: Controlled Intervention Studies, Observational Cohorts and Cross‐Sectional Studies, and Case–Control Studies. All articles were independently assessed by two reviewers (PdB and WEK) rating each domain as ‘yes’, ‘no’, ‘not applicable’, or ‘not reported’. The overall rating of each study could be ‘good’, ‘fair’, or ‘poor’. Any disagreement on the bias risk assessment between the two reviewers was resolved by discussion.

### 2.4 Statistical analysis

Statistical analyses were performed using the Cochrane Collaboration's software for preparing and maintaining Review Manager 5.4.1.. A quantitative synthesis (meta‐analysis) for generating an estimate on the effect size was possible. This meta‐analysis was conducted to the primary outcome: TIMP‐1 concentration (ng/mL) (mean ± SD) compared between periodontitis/ gingivitis patients and healthy individuals. In case in a study varying degrees of periodontal disease were monitored, the most severe condition was included. When in a study both chronic and acute periodontitis patients were monitored, data from the chronic patients were included in the analysis. Because of lack of identity between the included studies, the random‐effects model was used to perform the meta‐analysis.^19^ *I^2^*‐values higher than 50% were considered as indicative of substantial heterogeneity. *P*‐values less than 0.05 were considered as statistically significant.

## 3 ETHICAL REVIEW

This study was approved by the ACTA Ethics Committee (registration number 2020113).

## 4 RESULTS

### 4.1 Summary of the literature search and description of the included studies

The literature screening and selection process is presented in Figure 1. The search strategy retrieved a total of 77 studies using the PubMed database and 245 studies upon searching the Web of Science database. After removal of duplicate records, the titles and abstracts of the remaining 252 records were screened on inclusion and exclusion criteria. In total, 219 records were removed from the study. Detailed reading of the full text of the remaining 33 articles led to the additional removal of 19 records. Six articles only determined MMP/ TIMP‐1 ratio, three articles did not investigate TIMP‐1, one did not investigate periodontal disease in combination with TIMP‐1, in three articles the measurement of TIMP‐1 concentrations was discontinued during the study, three articles investigated interventions, and three articles were rejected for other reasons. The authors of six of the remaining 14 articles were approached for additional data. Two of the corresponding authors provided extra data. The author of another study reported that the records of the studies no longer existed, and the authors of the remaining three studies did not respond to the request for additional data. Therefore, these four articles were also excluded, based on missing data. The 10 remaining articles were included in the study and used in the meta‐analyses.

The main characteristics of the included studies are described in Table 1. All selected studies were published between 2006 and 2019 and accounted for 1336 participants with a mean of 128 participants per study and an age range between 15 and 64 years. Of 597 patients suffering from periodontal disease salivary TIMP‐1 levels were measured. The included studies were executed in Turkey, Brazil, Finland, Sweden, Denmark, and the USA. Five studies used stimulated saliva as clinical fluid, three unstimulated saliva, and two studies GCF.

**TABLE 1:** List of the included studies

Author, year	Country	n (Total)	n (P/NP)	Gender (P/NP)	Age (mean or range) (P/NP)	Used reference standard criteria	Biological sample	Detection method (TIMP−1)	Study design
Emingil et al., 2006 ^21^	Turkey	60	40/20	♂ 25/9 ♀ 15/11	41.5/27.4	*Periodontitis*: CAL ≥5 mm, PD ≥6 mm in multiple sites of all four quadrants of the mouth. *Gingivitis*: BOP ≥10% *Control*: no BOP, no ABL observed in radiographs, PD <3 mm.	GCF	ELISA	Case–Control Study
Marcaccini et al., 2010^23^	Brasil	42	27/15	♂ 10/5 ♀ 17/10	44.1/44.9	*Periodontitis*: ≥2 teeth with PD ≥5 mm, CAL ≥6 mm, evidence of ABL observed in radiographs. *Control*: no BOP, no ABL observed in radiographs, PD <3 mm.	GCF	ELISA	Controlled Intervention Study
Gürsoy et al., 2010 ^5^	Finland	106	40/66	♂ 27/22 ♀ 13/44	50.7/48.6	*Periodontitis*: ≥14 teeth with PPD ≥4 mm. *Control*: No teeth with PPD ≥4 mm.	SS	ELISA	Cross‐Sectional Study
Buduneli et al., 2011 ^36^	Turkey	32	15/17	NR	NR/35.4	*Periodontitis*: ≥1 pocket in each quadrant with PD ≥5 mm and CAL ≥6 mm. *Control*: systemic and periodontal healthy.	SS	ELISA	Cross‐Sectional Study
Rathnayake et al., 2012^22^	Sweden	352	49/303	NR	64.4/42.6	*Periodontitis*: Mild (ABL >one‐third of the root length in <30% of sites) to severe (ABL >one‐third of the root length in >30% of sites) *Control*: No ABL observed in radiographs.	SS	ELISA	Cross‐Sectional Study
Meschiari et al., 2013^10^	Brazil	42	23/19	NR	NR	*Periodontitis*: ≥2 teeth with PPD ≥5 mm, CAL ≥6 mm and evidence of ABL observed in radiographs. *Control*: periodontal healthy subjects.	SS	ELISA	Controlled Intervention Study
Nizam et al., 2014 ^20^	Turkey	36	18/18	♂ 10/11 ♀ 8/7	42‐61/26–63	*Periodontitis*: ≥4 teeth in each jaw PD ≥5 mm, CAL ≥4 mm, ABL ≥50% in at least in two quadrants, BOP >80%. *Control*: no BOP, no ABL observed in radiographs.	US	ELISA	Case–Control Study
Morelli et al., 2014 ^37^	USA	67	34/33	♂ 14/10 ♀ 20/23	34.4/30.3	*Periodontitis*: ≥1 site with PD >3 mm, BOP >50%. *Gingivitis*: all PD <3 mm, BOP ≥10%. *Control*: all PD <3 mm, BOP <10%.	US	Bioplex Multiplex system	Observational Cohort Study
Lahdentausta et al., 2018^9^	Finland	481	285/196	♂ 194/120 ♀ 91/76	64.1/62.4	*Periodontitis*: Mild (ABL in cervical third of the root) to severe (ABL in the apical third of the root) and PPD ≥4 mm in ≥4 sites. *Control*: periodontal healthy, gingivitis and edentulous patients.	SS	ELISA	Cross‐Sectional Study
Nascimento et al., 2019^8^	Denmark	84	42/42	NR	18–35	*Experimental gingivitis*: ≥20 teeth in each jaw, PD ≤ 4 mm, CAL ≤ 2 mm.	US	ELISA	Observational Cohort Study

*Abbreviations*: *ABL*: *Alveolar bone loss*, *BOP*: *bleeding on probing*, *CAL*: *clinical attachment level*, *GCF*: *gingival crevicular fluid*, *NP*: *non*‐*periodontal disease*, *NR*: *not reported*, *P*:*Periodontal disease*, *PD*: *pocket depth*, *PPD*: *periodontal probing depth*, *SS*: *Stimulated saliva*, *US*: *unstimulated saliva*.

The reported TIMP‐1 outcome and main conclusions of the included studies are described in Table 2. The range of TIMP‐1 concentrations varied considerably between the included studies, from 0.32 ± 0.15 ng/mL to 719 ± 24 ng/mL in periodontitis/ gingivitis patients, and from 0.37 ± 0.20 ng/mL to 721 ± 24 ng/mL in healthy individuals (mean ± SD) (Table 2). This variance was not directly related to type of oral fluid investigated, sample handling, or study population (Table 1).

**TABLE 2:** Reported outcome for TIMP‐1 and periodontal disease

Author, year	Biological sample	TIMP−1 (ng/mL)		
		Periodontal disease (mean ± SD (n))	Control (mean ± SD (n))	Results and conclusions on TIMP−1 as biomarker for periodontal disease
Emingil et al., 2006^21^	GCF	P: 0.56 ± 0.33 (20) G: 0.32 ± 0.15 (20)	0.37 ± 0.20 (20)	Total amounts of TIMP−1 in GCF were significantly higher in the periodontitis and gingivitis group compared to the healthy group (*p *< 0.0001). The concentration of TIMP−1 in GCF was comparable to that of the healthy group (*p *= .074).
Marcaccini et al., 2010^23^	GCF	103 ± 63 (27)*	74 ± 47 (15)*	No difference in TIMP−1 levels between the groups at baseline, or after therapy. MMP−8/ TIMP−1 ratio was significantly higher in the periodontitis group compared to the healthy controls at baseline (*p *= .03). Periodontal treatment of the periodontitis patients resulted in a significantly lower MMP−8/TIMP−1 ratio (*p *= .001).
Gürsoy et al., 2010^5^	SS	61 ± 68 (40)	110 ± 72 (66)	TIMP−1 concentration in stimulated saliva is significantly lower (*p *= .001) in the periodontitis group than in the control group.
Buduneli et al., 2011^36^	SS	11 ± 5 (15)	9,6 ± 2,8 (17)	TIMP−1 levels between healthy controls, non‐Acute Myocardial Infarction (AMI) and AMI patients significantly different (*p *= .001). No statements on the comparison of healthy controls with non‐AMI periodontitis patients.
Rathnayake et al., 2012^22^	SS	264 ± 175 (49)	268 ± 206 (303)	The difference in TIMP−1 concentrations between healthy controls and periodontitis patients is not significant. MMP−8/TIMP−1 ratio is significantly higher in periodontitis patients than in the controls.
Meschiari et al., 2013^10^	SS	70 ± 111 (23)*	83 ± 127 (19)*	TIMP−1 concentration in stimulated whole saliva is not significantly different between healthy patients and periodontitis patients.
Nizam et al., 2014^20^	US	82 ± 62 (18)	298 ± 208 (18)	The salivary TIMP−1 concentration was significantly lower in the periodontitis group than in the control group (*p*<0.001). The ratio of MMP−8/ TIMP−1 was significantly higher in the periodontitis group than in the control group (*p *< .001).
Morelli et al., 2014^37^	US	P: 717 ± 24 (34) G: 719 ± 24 (34)	721 ± 24 (33)	A significant increase in salivary TIMP−1 concentrations from baseline to peak induction in all groups (*p *< .001). No significant change in MMPs/ TIMPs ratio. No significant difference between the healthy group and the periodontitis or gingivitis group at baseline (*p *= .15).
Lahdentausta et al., 2018^9^	SS	177 ± 116 (285)	212 ± 122 (196)	No significant difference in salivary TIMP−1 concentrations between healthy controls and periodontitis patients without acute coronary syndrome (ACS).
Nascimento et al., 2019^8^	US	G: 452 ± 300 (42)	543 ± 430 (42)	TIMP−1 levels in unstimulated saliva are positively associated with gingival inflammation to the similar magnitude as MMP−8. TIMP−1 concentrations were lower on day 35 of the gingivitis study than at the start of the experimental gingivitis study but no significant difference was found.

*Abbreviations*: *G*: *Gingivitis patients*, *GCF*: *gingival crevicular fluid*, *P*: *Periodontitis patients*, *SS*: *Stimulated saliva*, *US*: *Unstimulated saliva*. * Data provided by authors.

Among the 10 included studies, a wide variety of conclusions was presented. In seven studies, TIMP‐1 concentrations were lower in patients with periodontal disease than in healthy individuals, of which two found a significant difference.^5^, ^20^ In three studies, the TIMP‐1 values were higher in patients with periodontal disease compared to the healthy individuals, of which one study found a significant difference.^21^

Three studies found that the MMP‐8/ TIMP‐1 ratio was significant higher in periodontitis patients^20^, ^22^, ^23^ (Table 2). The increase in MMP‐8/ TIMP‐1 ratio in these studies was predominantly related to increased salivary MMP‐8 levels in periodontitis patients and not necessarily to decreased TIMP‐1 concentrations in saliva. Only Nizam and co‐workers observed a significant decrease in TIMP‐1 level, whereas all three articles found a significant increase in MMP‐8 concentration (Table 2).

### 4.2 Quality assessment

The methodological quality of the 10 included studies was analyzed through the use of tools from the National Heart, Lung, and Blood Institute (NHLBI). Some of the items of the quality assessment tool were defined ‘not reported’. Because not all these items had a relation to the focus of this study, TIMP‐1 as biomarker for periodontal disease, the outcome of these items weighted less in the assessment of study quality. Among the six observational cohort studies and studies with a cross‐sectional design, five were rated good and one was judged fair (Table 3). Both control intervention studies were rated fair (Table 4), due to the high number of ‘not reported’ items. None of the two control intervention studies applied randomization of the study population. Of the two case control studies, one rated good whereas the other study was judged fair (Table 5). The difference in quality is mainly due to lack of correction for potential confounders and differences in recruitment populations between the periodontal disease patients and control group participants.

**TABLE 3:** Quality assessment tool for Observational Cohort and Cross‐Sectional studies

	Lahdentausta et al., 2018^9^	Gürsoy et al., 2010^5^	Buduneli et al., 2011^36^	Nascimento et al., 2019^8^	Morelli et al., 2014^37^	Rathnayake et al., 2012^22^
1. Was the research question or objective in this paper clearly stated?	YES	YES	YES	YES	YES	YES
2. Was the study population clearly specified and defined?	YES	YES	YES	YES	YES	YES
3. Was the participation rate of eligible persons at least 50%?	YES	NO	NR	NR	NR	NO
4. Were all the subjects selected or recruited from the same or similar populations (including the same time period)? Were inclusion and exclusion criteria for being in the study pre‐specified and applied uniformly to all participants?	YES	YES	YES	YES	YES	YES
5. Was a sample size justification, power description, or variance and effect estimates provided?	NR	NR	NR	NR	YES	NR
6. For the analyses in this paper, were the exposure(s) of interest measured prior to the outcome(s) being measured?	YES	YES	YES	YES	YES	YES
7. Was the timeframe sufficient so that one could reasonably expect to see an association between exposure and outcome if it existed?	NA	NA	NA	YES	YES	NA
8. For exposures that can vary in amount or level, did the study examine different levels of the exposure as related to the outcome (e.g., categories of exposure, or exposure measured as continuous variable)?	NO	NO	YES	YES	YES	YES
9. Were the exposure measures (independent variables) clearly defined, valid, reliable, and implemented consistently across all study participants?	YES	YES	YES	YES	YES	YES
10. Was the exposure(s) assessed more than once over time?	NO	NO	NO	YES	YES	NO
11. Were the outcome measures (dependent variables) clearly defined, valid, reliable, and implemented consistently across all study participants?	YES	YES	YES	YES	YES	YES
12. Were the outcome assessors blinded to the exposure status of participants?	NO	NO	NO	NO	NO	NO
13. Was loss to follow‐up after baseline 20% or less?	NA	NA	NA	YES	YES	NA
14. Were key potential confounding variables measured and adjusted statistically for their impact on the relationship between exposure(s) and outcome(s)?	YES	YES	YES	NO	YES	YES
Results	GOOD	FAIR	GOOD	GOOD	GOOD	GOOD

*Abbreviations*: *NA*: *Not applicable*, *NR*: *Not reported*.

**TABLE 4:** Quality assessment tool for Controlled Intervention Studies

	Meschiari et al., 2013^10^	Marcaccini et al., 2010^23^
1. Was the study described as randomized, a randomized trial, a randomized clinical trial, or an RCT?	NO	NO
2. Was the method of randomization adequate (i.e., use of randomly generated assignment)?	NO	NO
3. Was the treatment allocation concealed (so that assignments could not be predicted)?	NA	NA
4. Were study participants and providers blinded to treatment group assignment?	NA	NA
5. Were the people assessing the outcomes blinded to the participants’ group assignments?	YES	NR
6. Were the groups similar at baseline on important characteristics that could affect outcomes (e.g., demographics, risk factors, and co‐morbid conditions)?	YES	YES
7. Was the overall drop‐out rate from the study at endpoint 20% or lower of the number allocated to treatment?	NO	YES
8. Was the differential drop‐out rate (between treatment groups) at the endpoint 15% points or lower?	NO	YES
9. Was there high adherence to the intervention protocols for each treatment group?	YES	YES
10. Were other interventions avoided or similar in the groups (e.g., similar background treatments)?	NR	NR
11. Were outcomes assessed using valid and reliable measures, implemented consistently across all study participants?	YES	YES
12. Did the authors report that the sample size was sufficiently large to be able to detect a difference in the main outcome between groups with at least 80% power?	NR	NR
13. Were outcomes reported or subgroups analyzed pre‐specified (i.e., identified before analyses were conducted)?	NR	YES
14. Were all randomized participants analyzed in the group to which they were originally assigned, that is, did they use an intention‐to‐treat analysis?	NR	NR
Results	FAIR	FAIR

*Abbreviations*: *NA*: *Not applicable*, *NR*: *Not reported*.

**TABLE 5:** Quality assessment tool for Case–Control Studies

	Nizam et al., 2014^20^	Emingil et al., 2006^21^
1. Was the research question or objective in this paper clearly stated and appropriate?	YES	YES
2. Was the study population clearly specified and defined?	YES	YES
3. Did the authors include a sample size justification?	YES	NR
4. Were controls selected or recruited from the same or similar population that gave rise to the cases (including the same timeframe)?	YES	NO
5. Were the definitions, inclusion and exclusion criteria, algorithms or processes used to identify or select cases and controls valid, reliable, and implemented consistently across all study participants?	YES	YES
6. Were the cases clearly defined and differentiated from controls?	YES	YES
7. If less than 100% of eligible cases and/or controls were selected for the study, were the cases and/or controls randomly selected from those eligible?	NR	NR
8. Was there use of concurrent controls?	NO	NO
9. Were the investigators able to confirm that the exposure/risk occurred prior to the development of the condition or event that defined a participant as a case?	YES	YES
10. Were the measures of exposure/risk clearly defined, valid, reliable, and implemented consistently (including the same time period) across all study participants?	YES	YES
11. Were the assessors of exposure/risk blinded to the case or control status of participants?	NA	NA
12. Were key potential confounding variables measured and adjusted statistically in the analyses? If matching was used, did the investigators account for matching during study analysis?	YES	NR
Results	GOOD	FAIR

*Abbreviations*: *NA*: *Not applicable*, *NR*: *Not reported*.

### 4.3 Meta‐analysis

**FIGURE 2:**
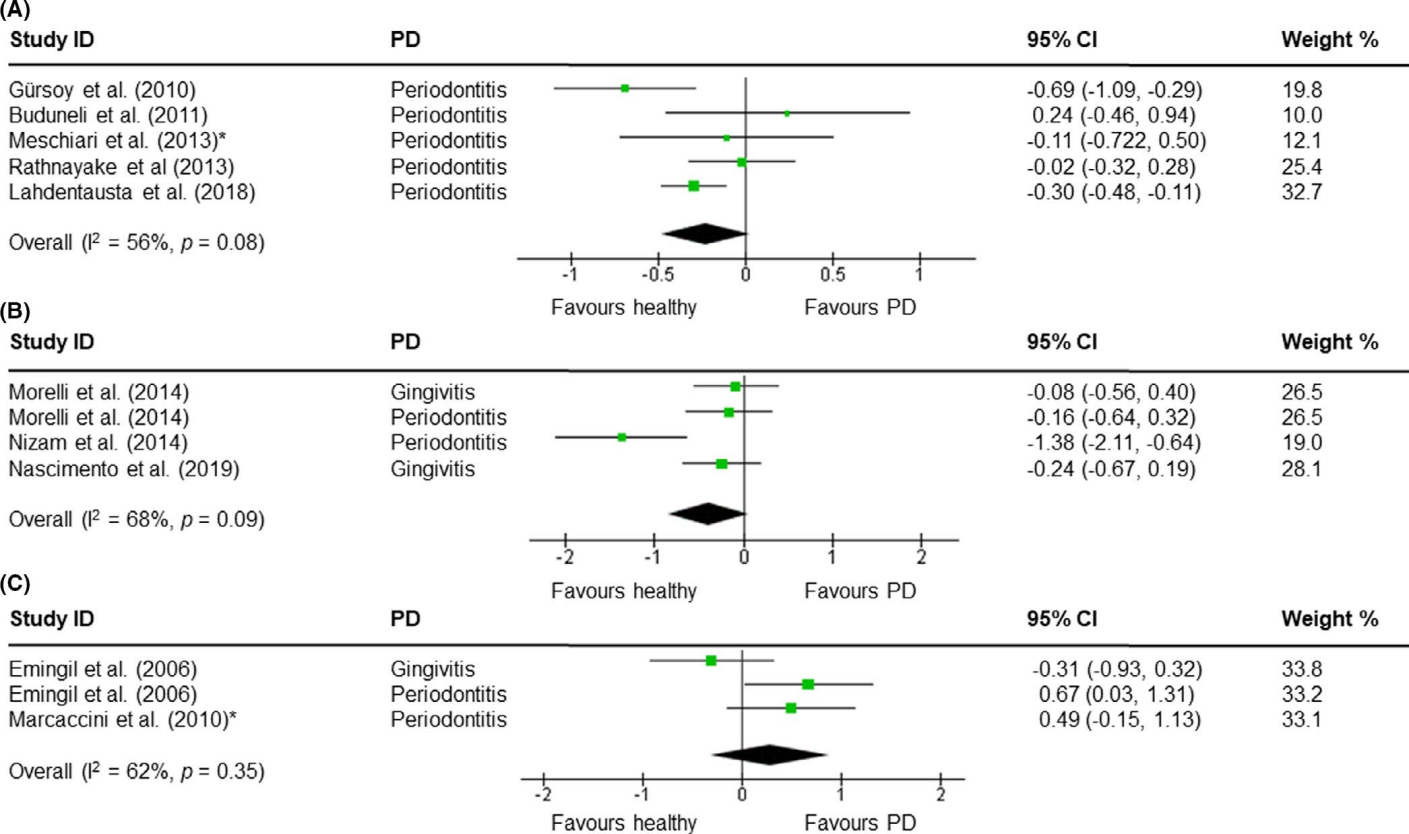
Forest plots of the meta‐analysis. Comparison of TIMP‐1 levels in stimulated saliva (A), unstimulated saliva (B), and GCF (C) from periodontitis/ gingivitis patients and healthy individuals using a random‐effects model. Periodontal disease (PD), confidence interval (CI), and heterogeneity (*I^2^*), * data provided by authors

For the meta‐analysis, the 10 included studies were grouped on oral fluid used: stimulated saliva, unstimulated saliva, and GCF. For each oral fluid, TIMP‐1 levels were compared between periodontitis/ gingivitis patients and healthy individuals. No statistically significant difference in TIMP‐1 levels in stimulated saliva was observed between healthy individuals (n = 601) and participants with periodontitis (n = 412) (*p* = .08) (Figure 2A). Three studies showed a higher salivary level of TIMP‐1 in healthy individuals, one study showed a higher level of TIMP‐1 in participants with periodontal disease, and one study showed no difference in TIMP‐1 levels between healthy participants and participants with periodontal disease. The heterogeneity of these studies was moderate (56%). Also, for unstimulated saliva, no statistically significant difference in salivary TIMP‐1 concentration was observed between healthy individuals (n = 126) and periodontitis/ gingivitis patients (n = 128) (*p *= .09) (Figure 2B). Three studies showed a higher level of TIMP‐1 in healthy participants, and in one study, no difference in TIMP‐1 levels between healthy participants and periodontitis/ gingivitis patients was observed. The heterogeneity between these studies was relatively high (68%). No statistically significant difference was observed between the periodontitis/ gingivitis (n = 67) and healthy (n = 35) groups (*p* = .35) when GCF was used as diagnostic fluid (Figure 2C). Both studies showed a higher level of TIMP‐1 in periodontitis patients, and the heterogeneity between the two studies was relatively high (62%).

## 5 DISCUSSION

This systematic review with subsequent meta‐analysis systematically evaluated levels of TIMP‐1 in oral fluids of periodontitis/ gingivitis patients and healthy individuals of 10 independent studies from six different countries. In general, our results showed that TIMP‐1 levels do not differ significantly between periodontitis/ gingivitis patients and healthy individuals in both saliva and GCF.

Several studies have shown that salivary concentrations of MMP‐8 and MMP‐9, the two MMPs regulated by TIMP‐1, have a high diagnostic value for periodontal disease.^24^, ^25^, ^26^ This has resulted in the suggestion that TIMP‐1, like MMP‐8 and MMP‐9, could serve as a diagnostic biomarker for periodontal disease. However, the included studies on the potential use of TIMP‐1 as biomarker for periodontal disease varied greatly in results (Table 2). This is reflected in the outcome of the meta‐analysis which showed that the difference in TIMP‐1 concentration between healthy participants and patients with gingivitis or periodontitis did not reach statistical significance, and the heterogeneity between the studies was relatively high (Figure 2A‐C). Of the 10 included studies, only two reported a significant difference between periodontitis/ gingivitis patients and healthy individuals.^5^, ^20^ These two studies used different methods for sample collection, which indicates that these changes in salivary TIMP‐1 concentrations are not related to the type of collection. Additionally, we could not find significant differences concerning study population (with regard to age, gender, and inclusion of smokers) and severity or phase (acute/ chronic) of the periodontal disease between the studies that reported a significant change in TIMP‐1 and the included studies who did not. The high SD values indicate that there is a large variation in TIMP‐1 concentration in both the periodontal disease group as in the control group (Table 2), which suggests that other confounders might be present.

A confounder known to influence TIMP‐1 production is smoking; a high number of pack years and recent cessation are associated with increased salivary TIMP‐1 levels.^12^ The studies included in our systematic review only reported inclusion or exclusion of smoking individuals but did not provide information on pack years and recent cessation. Furthermore, TIMP‐1 has numerous key roles in important biological processes. For example, TIMP‐1 plays an important role in adipocyte differentiation.^27^ Therefore, body weight might be a variable which affects the TIMP‐1 concentration in oral fluids. This suggestion is supported by the results of Caimi and co‐workers which show that in overweight and obese individuals’ serum TIMP‐1 levels are increased compared to individuals with a healthy body weight.^28^, ^29^ Another function of TIMP‐1 is the regulation of bone formation, by stimulating the bone‐resorbing activity of osteoclasts.^27^ Increased TIMP‐1 concentrations are indicative of an altered bone homeostasis, and associated with diseases like osteoporosis and rheumatoid arthritis (RA).^30^, ^31^, ^32^ None of the studies included in the current review reported that they defined osteoporosis, post‐menopausal woman (a group more susceptible to osteoporosis), and RA as exclusion factors. Additionally, other studies have shown that physical exercise, which is known to stimulate bone resorption, leads to an increase in serum TIMP‐1 level (and increase in MMP‐8 and MMP‐9) suggesting that degree of exercise might be another potential confounder for TIMP‐1 concentration in oral fluids.^29^, ^33^ All these studies measured TIMP‐1 levels in serum and not in saliva. However, during periodontal inflammation serum proteins leak into the oral cavity.^34^ Therefore, it is possible that the contribution by elevated TIMP‐1 levels in serum of RA, osteoporosis, exercise, and overweight/obese individuals outweighs the decreased salivary TIMP‐1 values associated with periodontal disease.

Important criteria for a good biomarker are validity, reliability, and consistency.^35^ Whereas TIMP‐1 plays a role in a broad set of biological processes, its concentration shows a wide variation among healthy individuals which affects the consistency and reliability of TIMP‐1 as a biomarker. This is confirmed by the results presented in this systematic review with meta‐analysis in which no significant changes in TIMP‐1 concentrations in oral fluids were found between periodontal disease and healthy individuals. In conclusion, TIMP‐1 is no reliable biomarker for screening and diagnostic purposes of periodontal disease.

## CONFLICT OF INTEREST

The authors declare no conflicts of interest related to this study.

## AUTHOR CONTRIBUTIONS

PdB and WEK contributed to study conception and design and to the collection and data interpretation. PdB, HSB, and WEK contributed to statistical analysis and data interpretation. PdB, HSB, FB, and WEK contributed to data interpretation. All authors contributed to the manuscript draft and to critically revise the manuscript.

## Supporting information

Appendix S1

Table S1

## Data Availability

Not applicable.
