# Supplementary material for: Is TIMP‐1 a biomarker for periodontal disease? A systematic review and meta‐analysis
Source: J Periodontal Res. 2021 Dec 1;57(2):235–45. doi: 10.1111/jre.12957 (PMC9299605; doi:10.1111/jre.12957)
Supplement: Supplementary file 1 — Appendix S1 [file JRE-57-235-s002.docx]

**Appendix S1.** PubMed/ MedLine and Web of Science search strategies.

*PubMed/ MedLine*

1. SEARCH TERMS: ("periodontitis"[MeSH Terms]) OR ("periodontitis"[All Fields]) OR ("periodontal pocket"[All Fields]) OR ("gingivitis"[MeSH Terms]) OR ("gingivitis"[All Fields]) OR ("gingival pockets"[All Fields]) AND ("protease inhibitors"[MeSH Terms]) OR ("protease inhibitor"[All Fields]) OR ("protease inhibitors"[All Fields]) OR ("TIMP"[All Fields]) AND ("Saliva"[All Fields]) OR ("Saliva"[MeSH Terms]) OR ("GCF"[All Fields]) OR ("gingival crevicular fluids"[All Fields]) OR ("Crevicular Fluid Gingival"[All Fields]) OR ("fluid gingival crevicular"[All Fields]) OR ("fluids gingival crevicular"[All Fields]) OR ("gingival crevicular fluids"[All Fields]) OR ("Gingival Exudate"[All Fields]) OR ("exudate gingival"[All Fields]) OR ("exudates gingival"[All Fields]) OR ("Gingival Exudates"[All Fields])
2. FILTERS: “human”; English and Dutch language

*Web of Science*

1. SEARCH TERMS: (periodontitis OR gingivitis OR periodontal pockets OR gingival pockets) AND (protease inhibitors OR TIMP) AND (saliva OR GCF OR gingival crevicular fluids OR fluids gingival crevicular OR gingival exudate)
2. LIMITS: document type: articles
